# Ca^2+^ Signalling Induced by NGF Identifies a Subset of Capsaicin-Excitable Neurons Displaying Enhanced Chemo-Nociception in Dorsal Root Ganglion Explants from Adult *pirt-GCaMP3* Mouse

**DOI:** 10.3390/ijms22052589

**Published:** 2021-03-04

**Authors:** Gary W. Lawrence, Tomas H. Zurawski, J. Oliver Dolly

**Affiliations:** International Centre for Neurotherapeutics, Dublin City University, Collins Avenue, D09 V209 Dublin, Ireland; tom.zurawski@dcu.ie (T.H.Z.); oliver.dolly@dcu.ie (J.O.D.)

**Keywords:** Ca^2+^ imaging, nerve growth factor, TRPV1, capsaicin, nociception

## Abstract

Nociceptors sense hazards via plasmalemmal cation channels, including transient receptor potential vanilloid 1 (TRPV1). Nerve growth factor (NGF) sensitises TRPV1 to capsaicin (CAPS), modulates nociceptor excitability and induces thermal hyperalgesia, but cellular mechanisms remain unclear. Confocal microscopy was used to image changes in intracellular Ca^2+^ concentration ([Ca^2+^]_i_) across neuronal populations in dorsal root ganglia (DRG) explants from *pirt-GCaMP3* adult mice, which express a fluorescent reporter in their sensory neurons. Raised [Ca^2+^]_i_ was detected in 84 neurons of three DRG explants exposed to NGF (100 ng/mL) and most (96%) of these were also excited by 1 μM CAPS. NGF elevated [Ca^2+^]_i_ in about one-third of the neurons stimulated by 1 μM CAPS, whether applied before or after the latter. In neurons excitable by NGF, CAPS-evoked [Ca^2+^]_i_ signals appeared significantly sooner (e.g., respective lags of 1.0 ± 0.1 and 1.9 ± 0.1 min), were much (>30%) brighter and lasted longer (6.6 ± 0.4 vs. 3.9 ± 0.2 min) relative to those non-responsive to the neurotrophin. CAPS tachyphylaxis lowered signal intensity by ~60% but was largely prevented by NGF. Increasing CAPS from 1 to 10 μM nearly doubled the number of cells activated but only modestly increased the amount co-activated by NGF. In conclusion, a sub-population of the CAPS-sensitive neurons in adult mouse DRG that can be excited by NGF is more sensitive to CAPS, responds with stronger signals and is further sensitised by transient exposure to the neurotrophin.

## 1. Introduction

The intricacies of how peripheral sensory nerve fibres elaborated from neurons clustered in dorsal root ganglia (DRG) and trigeminal ganglia (TG) efficiently sense an array of noxious stimuli are intriguing and pose research challenges for developing much-needed pain therapeutics that are effective and safe. Termed nociceptors because of being responsive to chemical, thermal, and mechanical hazards, they transduce signals utilising plasmalemmal cation channels and transfer information via ascending neurons in spinal cord and brainstem to the brain where pain is perceived. Although contributions are made by several of the transient receptor potential (TRP) protein sub-family [1,2], the best studied is a member activated by capsaicin (CAPS), a vanilloid from chili peppers. Cloning of the CAPS receptor established its identity as TRP vanilloid family member 1 (TRPV1), a non-selective cation channel with preference for Ca^2+^ that is activated by temperature (threshold 43 °C), extracellular protons plus numerous chemical deterrents [1,3]. In rat, it is expressed in just below half of the primary sensory neurons in both DRG and TG, where it functions in the hyper-sensitisation that underlies pain processing. Early investigations on TRPV1 largely focused on electrophysiological recordings from single sensory neurons isolated from rodent DRG (often neonates), and usually kept in culture. Although these yielded in-depth information on the current elicited in individual cells by CAPS, other studies have since distinguished sub-populations exhibiting distinct characteristics [4]. Additionally, there are valid questions about whether results obtained for neurons removed from ganglia, with disruption of intimate contacts with neighbours and other cell types, represent their true behavioural profiles. Both of these concerns have been avoided by utilising intact DRG explants from adult *pirt*-*GCaMP3* mice, expressing a fluorescent protein exclusively in nearly all peripheral sensory neurons with capability to sense intracellular Ca^2+^ concentration ([Ca^2+^]_i_) [5].

It has become pertinent to examine the influence of a natural promoter of pain, nerve growth factor (NGF), because of its known ability to sensitise sensory neurons [6,7,8,9,10,11]. This neurotrophin gets released from a variety of cell types following inflammation or injury [12]; hence, its levels are elevated in animal models of pain and inflammation, as well as in humans suffering from chronic pain. Additionally, administration of NGF leads to hyperalgesia and allodynia (amplified responses to noxious stimuli and pain induction by those that are normally innocuous, respectively) [13,14,15]. Accordingly, blockade of NGF signalling can alleviate nocifensive behaviour in rodent models and relieve symptoms of neuropathic or other pain types in patients [16,17]. The promotion of inflammatory hyperalgesia by NGF seems to be predominantly mediated by interaction with tropomyosin kinase receptor A (TrkA), which is expressed in about half of rat DRG neurons (DRGNs) [18], rather than its lower-affinity interaction with the more widely distributed pan neurotrophin p75 receptor [19,20,21]. NGF induces thermal hyperalgesia principally by sensitising TRPV1 [9,21]. The latter is expressed to variable levels in approximately half of the TrkA-positive neurons in adult rat DRG, but its mRNA has also been found at a lower level in isolectin IB4-binding, non-peptidergic neurons that were labelled for somatostatin [22]. In addition to long-term elevation by NGF of TRPV1 expression, acute effects on primary sensory neurons occur within minutes via at least three distinct mechanisms: it enhances the response of TRPV1 to CAPS and heat by (1) promoting channel opening, (2) attenuating desensitisation and (3) increasing trafficking of the channel from an intra-neuronal vesicular pool to the plasmalemma [9,23,24]. Consequently, the density of the channel on the cell surface is raised, so the amplitude of currents elicited by noxious stimuli and the accumulation of Ca^2+^ inside the sensitised neurons are both increased. Antagonism of SNARE-dependent exocytosis with a SNAP-25 peptide partially reduced NGF-evoked surface trafficking of the TRPV1 channel [25]. Likewise, inactivating SNAP-25 with a novel chimera of botulinum neurotoxins/E and/A in DRGNs abolished the elevation by tumour necrosis factor alpha (TNFα) of regulated exocytotic delivery to the plasmalemma of functionally active TRPV1 and TRP ankyrin family member 1 (TRPA1) [26]. In DRG and TG neurons cultured in the presence of NGF, at least part of the intracellular reserve of TRPV1 is located on large dense-core vesicles that mediate Ca^2+^-dependent calcitonin gene-related peptide release [27,28].

Due to about half of the population suffering from some form of pain and the existing treatments not being very effective and often with adverse side-effects, a need exists for more efficient, non-addictive pain therapeutics. Whilst only limited success has been reported for TRPV1 channel antagonists as analgesics, neutralising NGF antibodies are showing some promise (see [16,17]). In view of the increased relevance of NGF as a target for analgesics, its effects on large populations of sensory neurons and influence on their CAPS excitability were examined herein, by microscopic monitoring of [Ca^2+^]_i_ in intact DRG from *pirt-GCaMP3* mice. Exposure to NGF induced a slow-onset but persistent increase in [Ca^2+^]_i_ that was mostly restricted to a subset of CAPS-excitable neurons. In addition, this sub-population was more readily excited by CAPS and exhibited stronger [Ca^2+^]_i_ signals than cells that were not exposed or proved unresponsive to NGF. Thus, it is concluded that NGF-sensitive nociceptors in ganglia of healthy adult mouse represent a primed population acutely tuned to sense noxious environmental cues. Moreover, they are rapidly sensitised even further by acute exposure to NGF, a likely mechanism for the hyperalgesia associated with its heightened expression during inflammatory pain.

## 2. Results

### 2.1. NGF Induces a Slow but Persistent Increase in [Ca^2+^]_i_ Signals in a Sub-Population of CAPS-Excitable Neurons

**Figure 1:**
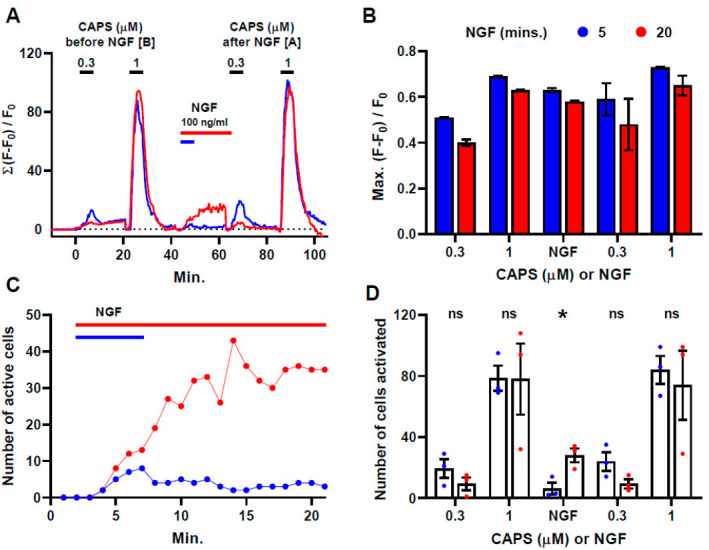
NGF evokes Ca2+ signals in a subset of CAPS-excitable cells. (**A**) Traces show increases in fluorescence intensity (summed from 3 experiments) in DRGNs exposed sequentially to CAPS for 5 min. (black bars, at concentrations indicated) and 100 ng/mL NGF for 5 (blue) or 20 min. (red). In the latter case, a 5 min. washout after the 20 min. exposure to NGF has been omitted from the figure to keep the subsequent responses to CAPS in register with those in the blue trace. Note that F_0_ was re-zeroed (see Section 4) before each addition of CAPS or NGF. Examples of fluorescence traces from individual DRGNs are presented in Supplementary Figure S1. (**B**) Shows the mean ± s.e.m. Max. fluorescence intensity increases in neurons that responded above threshold upon exposure to each stimulus, as indicated on abscissa, in DRG exposed to NGF for 5 (blue bars) or 20 min. (red bars). Significant differences were observed for 0.3 μM CAPS before NGF (*p* = 0.03), reflecting minor variation between experimental groups, and for 1 μM CAPS after NGF (*p* = 0.02) but this latter difference might be due to the high background fluorescence after 20 min. with NGF, which may interfere with the quantification of subsequent responses to CAPS. (**C**) Number of active cells counted in 1 min. intervals after the addition of NGF, plotted against time; data summed from 3 independent recordings for each NGF treatment. (**D**) Mean number of cells activated (± s.e.m., N = 3) over 20 min. after the addition of CAPS (at concentrations indicated), or NGF, in DRG exposed to the latter for 5 (blue) or 20 min. (red). Results obtained with either CAPS concentration are shown for a two-tailed Student’s *t*-test between groups exposed to NGF for 5 min. and those treated for 20 min; * *p* < 0.05; ns, not significant. Full statistical analysis is detailed in the Supplementary Table S1.

It is warranted to investigate how factors that promote pain may influence the activity of nociceptors in response to potentially harmful stimulants, represented by CAPS. Two experimental protocols were devised to test how sensitivity to NGF might be related to the robustness of responses to CAPS (Figure 1A), employing repeated stimulation of DRG so that signals elicited by various treatments could be compared within the same populations of neurons. This is advantageous because in many neurons the resting level of fluorescence before stimulation was too low to identify their presence. Both protocols began with a 5 min. exposure to 0.3 µΜ CAPS followed by 15 min. washout before a 5 min. treatment with 1 µΜ CAPS and 15 min. washout. The DRG were then acutely exposed to 100 ng/mL NGF for either 5 min. followed by 15 min. washout (blue trace) or 20 min. (red) followed by a 5 min. washout (this period is omitted from the trace to keep the following stimulations in register) before repeating the sequential application of 0.3 and 1 µΜ CAPS for 5 min. each with 15 min. washouts. Summed fluorescence was plotted against time (Figure 1A) for neurons exhibiting increments in signal intensity of at least 10 s.d. above a baseline (measured immediately before applying each stimuli). In accord with the literature [5,29], the traces both show the expected dose-dependent responses to CAPS, before and after exposure to NGF (Figure 1A); summed fluorescence was much higher for 1 µΜ than 0.3 µΜ CAPS largely due to a big increase in the number of cells excited by the higher concentration combined with a modest gain in signal intensity (Figure 1B). Although it is known that TRPV1 is essential for CAPS to elicit fluorescence changes in sensory neurons from *pirt-GCaMP3* mice [30], downstream activation of other channels or intracellular Ca^2+^ stores contributing to the signals evoked cannot be excluded. NGF alone also excited cells to produce Ca^2+^ signals that developed progressively over time (Figure 1A,C) such that the shorter incubation with NGF elicited far fewer active cells (Figure 1C,D) than the longer exposure. An accumulated total of 84 cells for recordings from 3 DRG were activated by NGF during a 20 min. exposure (Figure 1D) and all but 3 of these same cells (96%) were also excited by 1 µΜ CAPS at least once in the two exposures. Over the two application periods, 1 µΜ CAPS activated a total of 262 cells (234 and 219 the first and second time, respectively (Table 1); 195 were excited both times (Supplementary Table S3) and NGF excited 31% of these (81 of 262). By contrast, 5 min. with NGF excited far fewer neurons, 19 in total from 3 DRG, and two exposures to 1 µΜ CAPS evoked signals in 306 cells (not tabulated). Hence, a lower proportion was activated by the shorter treatment with NGF (19/306 = 6%) and, consequently, the low values in the corresponding part of the summed fluorescence trace. Notably, the mean intensity of signals in the responding cells was no lower than the level induced by 20 min. NGF and similar to that evoked by 1 μM CAPS (Figure 1B).

### 2.2. NGF Attenuates CAPS-Induced Tachyphylaxis Which Occurs in a Sub-Population of the DRGNs

**Figure 2:**
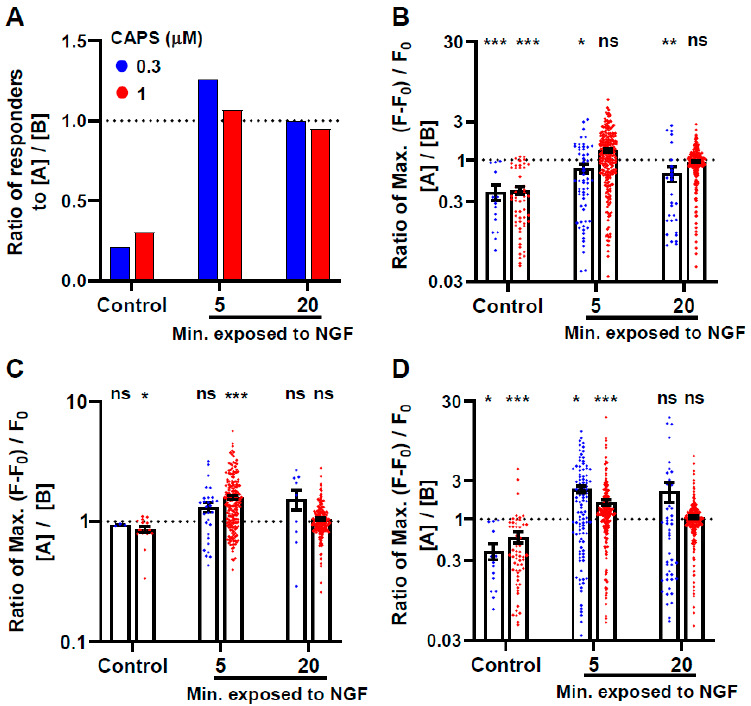
NGF attenuates CAPS tachyphylaxis. Response ratios (peak value obtained for second CAPS stimulation after [A]/peak value for first stimulation before [B] exposure to 100 ng/mL NGF) for the (**A**) number of responders or (**B**–**D**) Max. signal intensity evoked during the second exposure to 0.3 (blue) or 1 μM CAPS (red) divided by the values elicited the first time when the DRG were stimulated with the same concentration of agonist. In (**B**–**D**), the calculations were restricted to cells that conformed to the following criteria: (**B**) neurons that produced above threshold signals to 0.3 or 1 μM CAPS the first time each concentration was applied; note that both above and below threshold values elicited by the second stimulation with either agonist concentration were included in these analyses, (**C**) neurons excited above threshold both times the DRG were exposed to 0.3 or 1 μM CAPS, (**D**) all DRGNs excited at least one time by either the first or second stimulation with 0.3 or 1 μM CAPS; although the threshold criterion was used to identify the neurons to analyse, comparisons before and after NGF included values both above and below threshold. Paired two-tailed Student’s t-test were performed to compare the mean values of [A] and [B]; * *p* < 0.05; ** *p* < 0.01; *** *p* < 0.001; ns, not significant. Full statistical analysis and replicate numbers are detailed in Supplementary Tables S2–S4.

When a DRG was exposed twice to CAPS, as depicted in Figure 1A, but simply washed with recording buffer instead of treatment with NGF, far fewer neurons responded to the second round (Figure 2A) of agonist stimulation (6 and 19, respectively) in comparison to the first time either 0.3 (14 neurons) or 1 µΜ CAPS (54 cells) was applied (Supplementary Table S2). By contrast, there were only small differences in the numbers activated, by either 0.3 or 1 µΜ CAPS, before and after treatment with NGF for 5 or 20 min (Figure 2A). Likewise, in neurons excited by the first exposure to 1 µΜ CAPS, the Max. amplitude of evoked signals was very significantly lower (57%, *p* = 0.0004) for the second stimulation including many that declined to sub-threshold levels, but unchanged (*p* > 0.05) if the DRG were treated for 5 or 20 min with NGF in-between (Figure 2B, Supplementary Table S2). Such results are indicative of tachyphylaxis that was prevented by NGF. By contrast, NGF (either 5 or 20 min) only partially mitigated tachyphylaxis of signals evoked by 0.3 µΜ CAPS (Figure 2B, Supplementary Table S2). Even if the analysis was restricted only to neurons that responded (i.e., signals above threshold) both times the DRG were stimulated with 1 µΜ CAPS, the intensity of the second response was significantly reduced in the absence of NGF (~15%, *p* = 0.02), but was unchanged in those exposed to the growth factor for 20 min and actually increased ~19% (*p* < 0.0001) in ganglia treated with NGF for 5 min (Figure 2C and Supplementary Table S3). Similar trends were observed for 0.3 µΜ CAPS but did not reach significance. Acute (5 min) exposure to NGF enabled recruitment by 0.3 and 1 μM CAPS of 44 and 70 cells, respectively, which had not been excited prior to exposure to the growth factor. These figures were calculated by subtracting the number activated upon the first stimulation by CAPS (58 and 236; Supplementary Table S2) from the totals excited either time (102 and 306; Supplementary Table S4). Such increases indicate augmentation by NGF of signal intensity from below to above threshold in these cohorts. The latter seem to contribute to the maintenance of a large number of excited neurons and, thereby, the facilitation of average Max. intensity in the whole population of responding neurons (i.e., all DRGNs that responded at least once to 0.3 or 1 μM CAPS, respectively; Figure 2D, Supplementary Table S4). However, fewer new responders were recruited after 20 min. with NGF, 19 cells for 0.3 μM CAPS and 24 for 1 μM. NGF did not prevent tachyphylaxis completely; for example, of 236 cells that were excited by 1 μM CAPS before 5 min with NGF (Supplementary Table S2), only 183 responded a second time (Supplementary Table S3) indicating that 53 of them failed, possibly due to a lack of expressing appropriate neurotrophin receptors (see Section 3). In summary, tachyphylaxis occurred in a sub-population of CAPS-excitable DRGNs and was largely prevented by acute exposure to NGF which enhanced [Ca^2+^]_i_ signal intensity and, thereby, recruited more neurons and increased the average signal intensity in most (but not all) of the cells already responsive to the vanilloid. Responses to 1 μM CAPS were augmented more than those evoked by 0.3 μM and shorter (5 min.) exposure to NGF seemed more effective than longer (20 min), though this latter observation might be a consequence of persistent [Ca^2+^] signals after NGF which could interfere with the quantification of subsequent CAPS-evoked responses.

### 2.3. NGF-Excitable Cells Are More Responsive to CAPS

**Figure 3:**
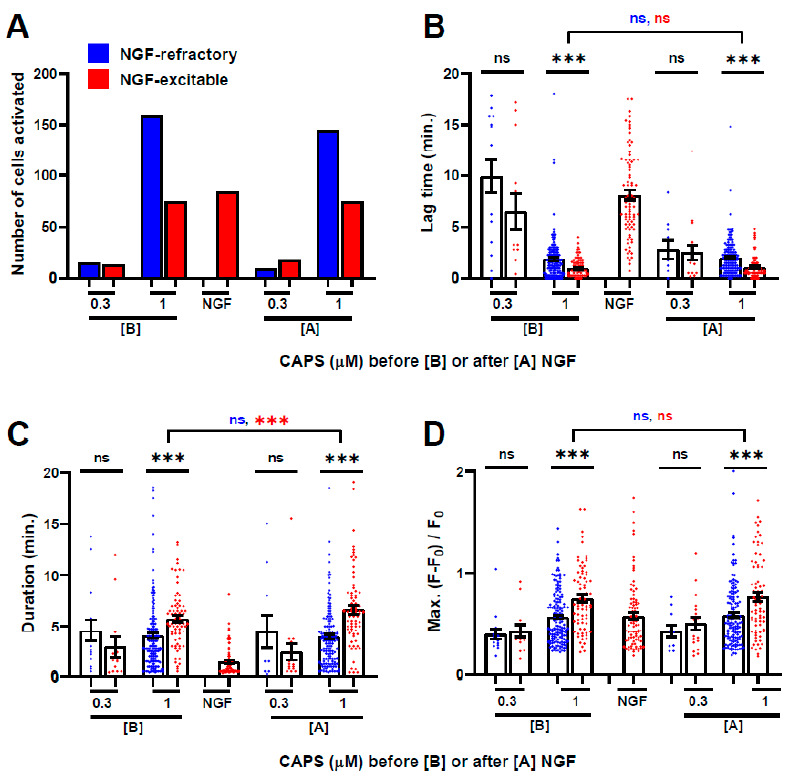
NGF-excitable cells exhibit more robust responses to CAPS. Further analysis was performed on DRG subjected to the protocol described for the red trace in Figure 1A. Cells were categorised according to whether or not they responded to 20 min. with NGF. (**A**) The number of NGF-excitable (red bars) and -refractory (blue bars) cells that responded to each treatment with the indicated CAPS concentrations applied before [B] or after [A] NGF. Analysis tools were applied to each sub-category to measure (**B**) Lag, (**C**) Duration and (**D**) Max. intensity, in all cases plotted as means ± s.e.m., with blue and red dots representing, respectively, values from individual cells assigned to the NGF-refractory and -excitable categories. Black asterisks represent *p* values derived by unpaired Student’s t-test between the NGF-excitable and –refractory cells within each CAPS stimulation group. Coloured asterisks show results from paired Student’s t-test for measurements of signals evoked by 1 μM CAPS before and after NGF, respectively, for each cell category; *** *p* < 0.001; ns, not significant. Full statistical analysis is described in Supplementary Tables S5 and S6.

Having identified an NGF-activatable sub-population of CAPS-excitable DRGNs, it became pertinent to examine whether that cohort reacts to the noxious stimulus differently from cells refractory to NGF (in terms of an absence of Ca^2+^ signals). The numbers of NGF-excitable and -refractory neurons activated by 0.3 µΜ CAPS were similar regardless of whether it was applied before (13 excitable and 15 refractory) or after 20 min exposure to NGF (18 excitable, 10 refractory; Figure 3A). Far more were excited by 1 µΜ CAPS and this involved approximately twice as many NGF-refractory as -excitable neurons (159 vs. 75, respectively, before NGF, 144 vs. 75 after). Thus, larger proportions of NGF-excitable cells were activated by the lower concentration of CAPS (13/28 = 46% before NGF, 18/28 = 64% after) relative to the higher concentration (75/234 = 32% before NGF, 75/219 = 34% after) (Supplementary Table S5). To put it another way, NGF-excitable cells are more likely to be activated by low concentrations of CAPS than those refractory to NGF. In NGF-excitable cells, the Ca^2+^ signals induced by 1 µΜ CAPS before [B] and after [A] NGF had significantly shorter lag times ([B] 47%, *p* < 0.0001; [A] 45%, *p* < 0.0001; Figure 3B), longer durations ([B] 45%, *p* < 0.0001; [A] 69%, *p* < 0.0001; Figure 3C) and higher Max. intensity ([B] 32%, *p* < 0.0001, [A] 17%, *p* = 0.0008; Figure 3D) than in their NGF-refractory counterparts. By contrast, there was no significant difference in any of the parameters for signals evoked by 0.3 µM CAPS (Supplementary Table S5). Thus, NGF-excitable cells constitute a sub-population of CAPS-activated sensory neurons that respond more robustly to the algogen.

### 2.4. Brief Exposure to NGF Further Enhances CAPS-Induced Signals

**Figure 4:**
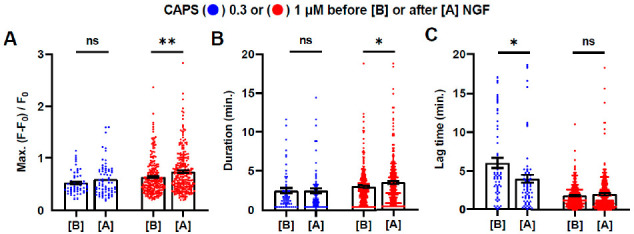
Brief exposure to NGF results in sensitisation to CAPS. Analysis tools were applied to signals evoked by 0.3 (blue dots) and 1 μM CAPS (red dots) before [B] and after [A] 5 min exposure to 100 ng/mL NGF (blue trace in Figure 1A) to measure (**A**) Max. intensity, (**B**) signal duration and (**C**) lag time. Asterisks represent significance between the measurements before and after NGF treatment, determined by Student’s t-test for unpaired samples, unequal variance; * *p* < 0.05; ** *p* < 0.01; ns, not significant. Full statistical analysis is detailed in Supplementary Table S7.

**Figure 5:**
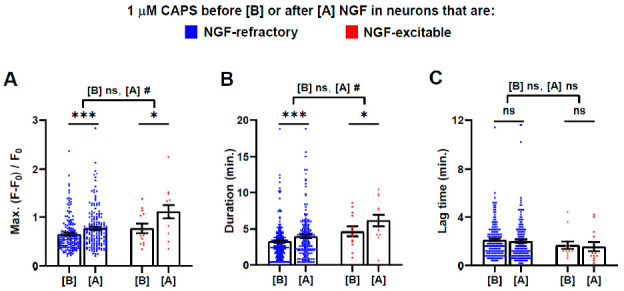
Acute sensitisation by NGF of responses to CAPS is most prevalent in cells that exhibit [Ca2+]i signals when the neurotrophin is applied. Neurons that responded to 1 μM CAPS both times when it was applied before and after 5 min exposure to NGF (blue trace in Figure 1A) were split into two groups depending on whether they additionally exhibited Ca^2+^ signals in response to NGF alone (red dots, *n* = 13 neurons from 3 DRG recordings) or did not (blue dots, *n* = 170). The analysis tools were applied to measure (**A**) Max. intensity, (**B**) signal duration and (**C**) lag time for the CAPS-evoked signals before [B] (on abscissa) and after [A] the treatment with NGF. Asterisks show significant differences within groups between responses before and after NGF treatment (Student’s *t*-test, paired samples; * *p* < 0.05; *** *p* < 0.001; ns, not significant). Hash tags display significant differences between NGF-refractory and -excitable cells, for responses to 1 μM CAPS before [B] and after [A] NGF (Student’s *t*-test, unpaired samples, unequal variance; # *p* < 0.05; ns, not significant). Detailed results of analysis are given in Supplementary Table S8.

Comparison of the Ca^2+^ signals induced by 0.3 or 1 µΜ CAPS before and after DRG were exposed to NGF for 5 min. revealed changes consistent with acute sensitisation of neurons by the growth factor (Figure 4). There were significant increases in the Max. intensity (16%, *p* = 0.002; Figure 4A) and duration (20%, *p* = 0.03; Figure 4B and Supplementary Table S7) of signals evoked by 1, but not 0.3, µΜ CAPS. By contrast, the lag was not significantly different (1.8 ± 0.1 min. before vs. 2.0 ± 0.1 min. after NGF, *p* = 0.24; Figure 4C). Although the lag before signal onset was significantly reduced (33%, *p* = 0.01) in the case of 0.3 µΜ CAPS (Figure 4C), a 48% reduction in lag was also observed in control cells and so this may be unrelated to NGF. Moreover, there were no significant changes in the Max. intensity (Figure 4A) and duration (Figure 4B) of 0.3 µΜ CAPS-evoked signals before and after NGF. The responses to 1 µΜ CAPS were analysed further by categorisation into NGF-responsive and -refractory sub-groups. After 5 min treatment with NGF, the Max. intensity of CAPS-evoked signals in NGF-responsive neurons was enhanced significantly (45%, *p* = 0.02) compared to before, and with respect to the signals in NGF-refractory cells (45% larger, *p* = 0.03; Figure 5A). Similarly, signal duration in NGF-excitable cells increased after NGF (32%; 0.03) and was greater in excitable cells than in NGF-refractory cells after NGF (55%, *p* = 0.02; Figure 5B). The smaller, but significant, increases after NGF exposure observed in intensity (20%, *p* < 0.0001; Figure 5A) and duration (25%, *p* < 0.0001; Figure 5B) for CAPS-evoked signals in NGF-refractory cells might be attributable to the concealed presence of NGF-responders due to the limited ability of 5 min with NGF to reveal them all (Figure 1C,D). Thus, it is even more remarkable that the small fraction of NGF-responders that were identified proved to be more active than the ‘NGF-refractory’ neurons. However, no significant difference in lag times were observed between NGF-excited and -refractory cells, or either group before or after NGF (Figure 5C). Unfortunately, the persistence of raised [Ca^2+^]_i_ in NGF-responders when exposed to the neurotrophin for 20 min precluded repeating this analysis for the group. To summarise, NGF-responsive cells are excited more robustly by 1 µM CAPS and become activated even more strongly after a brief exposure to the neurotrophin.

### 2.5. Prior Exposure to CAPS Is Not Necessary for the Activation by NGF of DRGNs: A Second Exposure to NGF Excites Even More Cells

**Figure 6:**
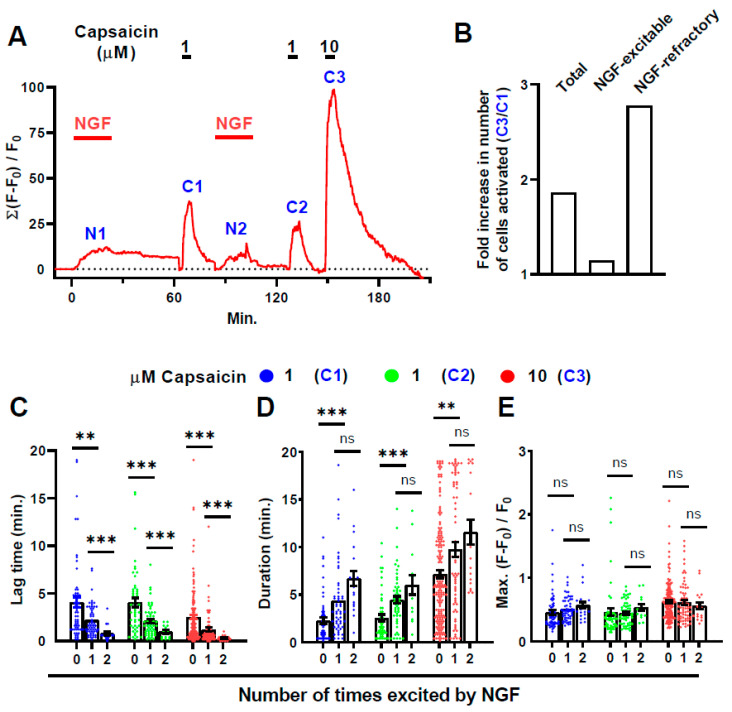
NGF evokes signals if applied before CAPS and excites a new cohort when re-applied afterwards; CAPS-induced signal strength correlates with responsiveness to NGF. (**A**) Summed increase in fluorescence from DRG treated, in the following order, with 100 ng/mL NGF (20 min followed by 40 min washout, [N1]); 1 μM CAPS (5 min, 15 min washout, [C1]); NGF for a second time (20 min, 20 min washout, [N2]); 1 μM CAPS again (5 min, 15 min washout, [C2]) and, finally, 10 μM CAPS (5 min, 35 min washout, [C3]). (**B**) Shows the relative increase in the number of active cells (number activated by 10 μM CAPS [C3]/number excited by 1 μM [C1]) calculated for all CAPS-excitable cells (Total) or only the subsets that were NGF-excitable and -refractory cells. (**C**–**E**) Neurons that responded during the first ([C1]; blue dots) or second exposure ([C2]; green dots) to 1 μM CAPS and those excited by 10 μM CAPS ([C3]; red dots) were sub-categorised according to whether they were excited both times (2, abscissa) on exposure to NGF (i.e., during [N1] and [N2]), only once (1, abscissa) by either application (i.e., [N1] or [N2]) or remained inactive both times (0, abscissa). Each sub-category was analysed for (**C**) lag time, (**D**) signal duration and (**E**) Max. intensity. Plotted data represents mean ± s.e.m. Asterisks indicate *p* values for Student’s *t*-tests compared between group 1 and the requisite data set in group 0, or between groups 1 and 2. ** *p* < 0.01; *** *p* < 0.001; ns, not significant. Detailed results are provided in Supplementary Tables S9 and S10.

In the next series of experiments, DRG were exposed to 100 ng/mL NGF for 20 min. followed by a 40 min washout before applying 1 µΜ CAPS (Figure 6A). As observed above, NGF provoked Ca^2+^ signals in about one third of the cells that respond to CAPS (47 of 135 cells = 35%, recordings from 3 different DRG; Table 2). As NGF was applied before CAPS, the signals can be attributed to a direct action of NGF. Due to the cells excited by NGF alone remaining fluorescent for the entire 40 min. washout (Figure 6A), their elevated level served as a baseline for the subsequent responses to CAPS; consequently, the increase in average fluorescence for the CAPS stimulus may be an underestimate. Re-application of NGF for a second time caused Ca^2+^ signals in 72 cells (Table 2). The majority of these ([72 − 21 = 51] out of 72 = 71%, Table 2) belonged to a new cohort that had not been activated by the first exposure to NGF though most of them (50 of 72 = 69%) had responded to 1 µΜ CAPS. There were 21 cells excited both times by NGF and all of them were also activated by CAPS.

### 2.6. Almost All the Cells Excited Only by 10, But Not 1, µΜ CAPS Were Recruited from a DRGN Population Refractory to NGF

After the DRG described above had been exposed to NGF a second time and the growth factor washed away, two further CAPS stimulations were applied i.e., 1 µΜ CAPS (5 min and 15 min washout) and, finally, 10 µΜ CAPS (5 min followed by washout until fluorescence returned to baseline). A slightly lower number of neurons was activated (121; Table 2) and intensity was reduced (summed from the activated cells) during the second exposure to 1 µΜ CAPS compared to the first time (Figure 6A) but subsequent application of the higher concentration resulted in a big increase in the number of activated neurons (251; Table 2) and, consequently, cumulative signal intensity (Figure 6A). Thus, the exposure to 10 µΜ CAPS activated 116 more neurons than the 135 evoked by the first-time exposure to 1 µΜ CAPS, a nearly 1.9-fold increase in the total number of excited cells (Figure 6B). However, most were recruited from the NGF-refractory sub-population (105 of 116, >90% [not tabulated]). Consequently, NGF-excitable cells constitute a smaller proportion of the cells excited by 10 µΜ CAPS (87/251; 35%) than those activated by 1 µΜ CAPS (76/135; 56%) (Table 2). On the other hand, most of the NGF-excitable cells (108 neurons from 2 stimulations) were activated by the first exposure to 1 μΜ CAPS (76/108; 70%), with only a few more recruited by 10 µΜ CAPS (87/108 = 81%), a 1.1-fold increase (87/76; Figure 6B), whereas nearly three times as many NGF-refractory cells were stimulated by 10 µΜ CAPS compared to the lower concentration (164/59 = 2.8; Figure 6B).

Comparison of the Ca^2+^ signalling characteristics between cells that were refractory to NGF, excited once, and those that responded both times re-affirmed that NGF-excitable cells respond to 1 µΜ CAPS with a faster onset (Figure 6C) and longer duration signals (Figure 6D). Moreover, it was shown by both these measures that cells which responded twice to NGF were even more excitable than those stimulated only once by the growth factor. Responses to 10 µΜ CAPS always appeared faster (Figure 6C) and lasted longer (Figure 6D) than signals induced by 1 µΜ CAPS, irrespective of how many times the cells had been excited by NGF. Furthermore, one-time NGF-excited cells responded faster and for longer to 10 µΜ CAPS than NGF-refractory cells (i.e., the 0 times excited group), with those that responded twice being the fastest and longest lasting of all (Figure 6C,D). There was a trend for higher Max. intensity responses to 1 µΜ CAPS in NGF- excitable cells, but these increments were not significant (Figure 6E) and there was no difference in Max. responses to 10 µΜ; note, however, that the Max. may be underestimated in the NGF-excitable cells due to the persistence of fluorescence after 20 min exposure to the neurotrophin.

Collectively, the data indicate that the NGF excitable cells are a sub-population of DRGNs that respond to CAPS much more robustly than other TRPV1-expressing cells and can be sensitised even further by short exposure to this neurotrophin. Acute exposure to NGF prevented tachyphylaxis, which occurred in a subset of CAPS excitable neurons, and attenuated desensitisation by augmenting the intensity and duration of Ca^2+^ signals. These findings provide new insights into how the detection of noxious chemicals is modulated by NGF, a factor released at low levels in healthy tissues but increased during injury or inflammation.

## 3. Discussion

It is well established that acute exposure to NGF sensitises a subset of TRPV1-expressing sensory neurons by enhancing surface expression and activity of the channel, but the impact on nociception in the context of DRGN populations is still poorly understood. To address such an important question, this study monitored Ca^2+^ signals evoked by different concentrations of the TRPV1 agonist CAPS, representing a noxious stimulus of increasing intensity. Advantageously, utilisation of the *pirt-GCaMP3* mouse facilitated the measurements exclusively over large populations of sensory neurons having preserved somatic organisation within DRG explants acutely removed from adult animals. A new method to identify and separately analyse neurons responsive to NGF is described, which demonstrated its direct induction of [Ca^2+^]_i_ signals, a feature not recognised previously. Notably, this unveiled a sub-population of DRGNs that are more robustly activated by CAPS in terms of increasing [Ca^2+^]_i_—faster activation, longer duration and higher intensity (Figure 3)—and sensitised even further by acute exposure to the neurotrophin (Figure 4 and Figure 5). There is an association between the latter and CAPS concentration, as heightened responses in the NGF excitable population were observed for the signals induced by 1 and 10, but not 0.3 μM CAPS. The involvement of [Ca^2+^]_i_ in NGF signalling has been a matter of considerable debate [31]; no evidence of NGF-inducing increases in [Ca^2+^]_i_ was found in Fluo-4 loaded DRGNs under conditions of acute exposure that caused its enhancement of CAPS-evoked signals [6,25]. A possible reason for the discrepancy with the current data is the relatively short exposure (2–8 min) to NGF used because, herein, the rise in [Ca^2+^]_i_ developed slowly and was highly dependent on its prolonged presence. Although only 6% of cells stimulated by 1 µM CAPS were excited by NGF within 5 min, reliability of this small change was reinforced by the responsible cohort of cells proving more sensitive to CAPS (Figure 4 and Figure 5). Furthermore, this value rose to 31% after 20 min (Table 1), and the average Max. intensity of signals in cells that did respond to 5 or 20 min with NGF was as high as in cells excited by 1 μM CAPS (Figure 1B). Applying NGF first for 20 min resulted in activation of 35% of the cells subsequently stimulated by 1 µΜ CAPS and a second 20 min exposure to NGF raised this to 56% (Table 2). Another possible explanation for the above-noted lack of reports on NGF-induced [Ca^2+^]_i_ signals is differences between mature neurons in acute DRG explants from adult mouse and immature cells from neonatal rats that were cultured initially in the presence of NGF, then starved of the growth factor for 24 h before experimentation [6,25]. Exposure to the neurotrophin for only 5 min was sufficient to attenuate tachyphylaxis and enhance CAPS-induced signalling, in accord with in vitro studies on dissociated cells, despite such a short period of exposure directly eliciting signals in only a few neurons. Therefore, [Ca^2+^]_i_ signals induced by NGF are probably not essential for sensitisation of TRPV1, but a modulatory role is possible. Moreover, neither the removal of extracellular Ca^2+^ nor intracellular chelators perturbs acute sensitisation by NGF of CAPS-evoked currents in vitro, and Ca^2+^ is not essential for the signalling pathways that have been implicated in the process [9,23,24,31]. The aforementioned longer treatments with NGF directly induced increases of [Ca^2+^]_i_, even if applied to the DRG before CAPS (Figure 6), so the signals induced were not just a consequence of NGF sensitising TRPV1 to the agonist or enabling re-opening of channels that had been activated previously by CAPS. Nevertheless, it is remarkable that NGF evoked [Ca^2+^]_i_ signals preferentially in CAPS-excitable neurons (Table 1 and Table 2) because TRPV1 mRNA has been detected in only about half of the rat DRG nociceptors that express TrkA, although the lower affinity p75 neurotrophin receptor is distributed more widely. As well as TRPV1, other plasmalemmal ion channels have been suggested to be downstream targets of NGF-activated signalling cascades, whilst activation of phospholipase C could theoretically induce release of Ca^2+^ from internal stores; so, further investigations are warranted into the mechanism for NGF increasing [Ca^2+^]_i_, including use of inhibitors for its receptors, but these are beyond the scope of the current study.

The extent of sensitisation by NGF of neurons varied greatly, in agreement with previous reports [6,10,11]. Even after 20 min exposure to NGF, tachyphylaxis persisted in a minority of the neurons ([234 − 195 = 39] out of 234; Table 1) such that they failed to respond above threshold to 1 µΜ CAPS a second time. By contrast, nearly all (93%) of those that were excited by both the first exposure to 1 µΜ CAPS, and again by 20 min treatment with NGF, did respond to the second stimulation by 1 µΜ CAPS without tachyphylaxis (Figure 2A); this may even be an underestimate due to persistent high background fluorescence in some cells after NGF (as noted in the Section 2). It was observed that many NGF-refractory cells were excited twice by 1 µΜ CAPS (Table 1), and even DRG that were not treated with NGF contained neurons that were activated by the vanilloid on both occasions with only minimal tachyphylaxis (Figure 2C). Thus, being receptive to NGF is not a prerequisite for neurons to retain sensitivity to 1 µΜ CAPS with minimal tachyphylaxis, but it is strongly associated with the latter. Notably, tachyphylaxis in the neuronal population was allayed by the recruitment of more CAPS-excitable cells; apparently, this lessened reductions in the number of responding neurons (Figure 1D) and the average signal intensity across the population (Figure 2D). It is not clear why a few neurons responded to the second stimulation with CAPS, despite failing to react to the first, but one possibility is that weak (below threshold) increases in [Ca^2+^]_i_ might facilitate subsequent signals. Moreover, in those exposed to NGF, signal intensity was enhanced from below to above threshold in many cases. Such new recruits would have been missed previously by protocols that used CAPS to screen for excitable cells, then measured changes only in the neurons identified thus but, clearly, these could be important for maintaining the integrated [Ca^2+^]_i_ response to noxious stimuli across the receptive population (Figure 1A). To summarise, tachyphylaxis of CAPS-evoked [Ca^2+^]_i_ increases was observed in many but not all neurons of lumbar DRG from adult *pirt-GCaMP3* mice, and in some of those only it was prevented by acute exposure to NGF. The ability of NGF to alleviate tachyphylaxis in certain CAPS-sensitive neurons, but not others, probably reflects expression of the NGF-receptor, TrkA, being limited to a sub-population of the DRGNs in adult rodents, including only about half of those that express TRPV1.

In contrast to its extensive attenuation of tachyphylaxis, it is noteworthy that, despite increasing signal duration [Figure 3C (c.f. 1[B] and 1[A])], acute exposure to NGF did not prevent desensitisation as signals ceased in many neurons well before CAPS was removed. As TRPV1 is essential for CAPS to produce increases in [Ca^2+^]_i_ in *GCaMP3* sensory neurons, such cessation must involve closure of these channels. NGF antagonises tachyphylaxis primarily by inducing the trafficking of more TRPV1 to the cell surface, whereas desensitisation may result from dephosphorylation of TRPV1 culminating in increased thresholds for CAPS; thus, NGF appears to replace desensitised TRPV1 rather than induce its re-sensitisation [29]. Although NGF can also modify the gating properties of TRPV1 by causing its phosphorylation, the relative importance of this is disputed [24,31]. Accordingly, in cultures of neonatal sensory neurons, a peptide inhibitor of exocytosis attenuated sensitisation to CAPS by NGF [25] and trafficking of TRPV1 and TRPA1 to the cell surface induced by TNFα, which is also known to sensitise sensory neurons, was abolished using botulinum neurotoxins to inactivate SNAREs required for membrane fusion [26,28].

In view of TRPV1 sensitisation modulating nociceptor thresholds and activity, it is an intriguing revelation that the most robust [Ca^2+^]_i_ responses to CAPS occur in a sub-population of DRGNs with which NGF interacts. This might be explained by a relatively high expression of TRPV1 mRNA in TrkA-positive, compared to non-peptidergic, neurons [22] and maybe relates to the link between the NGF-induced TRPV1 sensitisation and pain. The finding herein that NGF-responsive neurons from adult mice are more sensitive to CAPS suggests that endogenous NGF may also modulate nociceptor sensitivity in healthy tissues, despite expression levels being very low compared to earlier developmental stages or during inflammation. Accordingly, administration to rats of NGF-sequestering TrkA-conjugates resulted in a loss of sensitivity to noxious heat or CAPS, in addition to a near-complete abolishment of carrageenan-induced inflammatory hyperalgesia [32]. On the other hand, in both humans and rodents, exogenous NGF induced hyperalgesia that was dose-dependent [13,15]. Apparently, endogenous NGF concentrations set nociceptor thermal and chemical sensitivity in both healthy and disease states via sensitisation of TRPV1, consistent with reported adverse effects of anti-NGF therapies [17,33]. Nevertheless, clinical trials have resumed for the most promising anti-NGF monoclonal antibodies therapies with a low dose protocol [16,17].

## 4. Materials and Methods

### 4.1. Materials

The *pirt-GCaMP3* mice were a kind gift from Prof. X. Dong (Johns Hopkins University School of Medicine, Baltimore, MD, USA). All husbandry and experimental procedures were approved by the Research Ethics Committee of Dublin City University and the Irish Health Products Regulatory Authority (Project Authorisation no. AE19115/P020). CAPS and NGF (2.5 s) were purchased from Alomone (Jerusalem, Israel), and Liberase from Roche Diagnostics (Mannheim, Germany). All other chemicals were obtained from Merck (Arklow, Ireland).

### 4.2. Confocal Imaging of DRG Explants

Heterozygous adult (6–8 weeks) male or female *pirt-GCaMP3* mice were euthanised by cervical dislocation and L3 or L4 DRG dissected, with a few mm of the ventral and dorsal roots plus peripheral nerve trunk attached. Ganglia were collected in ice-cold dissection buffer (mM: CaCl_2_, 0.5; glucose, 11; KCl, 2.5; MgSO_4_, 10; NaHCO_3_, 26; NaH_2_PO_4_, 1.2; sucrose, 216) constantly gassed with a mixture of 95% oxygen: 5% carbon dioxide (O_2_/CO_2_). Within 10 min of dissection, the DRG were rinsed with artificial cerebrospinal fluid (aCSF; mM: CaCl_2_, 2; glucose, 11; KCl, 3.6; MgSO_4_, 1.2; NaCl, 117; NaHCO_3_, 25; NaH_2_PO_4_, 1.2) pre-gassed as above. The ganglion sheath was gently removed by a combination of mechanical peeling and 5 min. digestion at room temperature by Liberase™ (Roche Diagnostics; 13 units/mL in aCSF lacking the MgSO_4_) before placing the loosened DRG in a recording chamber (Warner Instruments, Holliston, MA, USA; PM1 and RC-26GLP; bath volume ~1 mL) under a tissue holder; washing ensued by superfusing at 2 mL/min. with normal aCSF containing 10 μg/mL bovine serum albumin and continuously gassed with O_2_/CO_2_ (aCSF-BOC) at ambient temperature (~22 °C). After 30 min the aCSF-BOC was warmed to 32 °C and washing continued for another 30 min. before starting the recordings. All subsequent experimental procedures and washes were performed at 32 °C with continuous superfusing of aCSF-BOC.

Confocal imaging was performed using a Zeiss Axio Examiner Z1 upright microscope with a 488 nm laser, using a 10 × magnification objective (NA 1.336) controlled by Zen 2008 (Carl Zeiss, Oberkochen, Germany). Z-stacks were configured to image the majority of the volume of each ganglion: 10 to 14 non-overlapping Z-planes of 25–30 μm thickness. Each confocal plane was scanned once every 10–15 s, depending on stack size. This protocol maximised the volume of the DRG imaged whilst the temporal resolution proved adequate to determine differences in activation lag time and duration of signals; with few exceptions, average lag times and durations were >1 min.

### 4.3. Image Analysis and Measurements of Signal Properties

Regions of interest (ROIs) where detectable changes in the intensity of fluorescence occurred were selected in time lapse movies of confocal stacks flattened using Image J (www.ImageJ.net; accessed on 25 February 2020). Occasionally, large diameter cells were seen to fluoresce in a slow cyclical manner that was obviously unrelated to DRG treatment protocols, so these were not included as ROIs. Movies were analysed in segments of 20 min (except initial baseline [10 min]) and ROI positions adjusted for minor shifts in tissue position and orientation. Pixel intensity within ROIs was measured using ImageJ and values transferred to Microsoft Excel^®^ (Office 365, Microsoft Corporation, St. Redmond, WA, USA) to perform calculations. In segments including experimental treatments (i.e., exposure to CAPS or NGF) the measurements made during the initial 2 min of each recording, representing a period just before the substance washed-in, were averaged to obtain base fluorescence values (F_0_). The intensity of emitted fluorescence (F) was measured for each ROI in all subsequent movie frames and the change in intensity relative to base values calculated for each time point using the formula (F − F_0_)/F_0_. The standard deviation (s.d.) in F was calculated for each ROI over the initial 10 min baseline recording. ROIs were considered to contain positive-responders if the averaged pixel intensity change, (F − F_0_)/F_0_, was greater than the base, F_0_, plus 10 × s.d. Only positive responders to each treatment were included in calculations of sum (Σ) of (F − F_0_)/F_0_, so this measurement increases with increments in either or both the number and the average fluorescence intensity of positive responders. Examples of the fluorescence signals elicited by CAPS or NGF are presented in Supplementary Figure S1.

To simplify analysis of complex and heterogeneous fluorescent signals from individual DRG neurons (DRGNs), peak analysis using GraphPad Prism 9 software (GraphPad Software, San Diego, CA, USA) was performed to measure: (1) Number of responders, a count of all the ROIs that exhibited at least one [Ca^2+^]_i_ signal above the threshold, (2) Lag, the time between test samples reaching the DRG recording chamber and the first [Ca^2+^]_i_ signal above threshold, (3) Duration, the accumulated period signal remained above threshold and (4) Max. (F − F_0_)/F_0_, the largest increment in fluorescence signal above baseline during a 20 min period immediately after introducing a stimulus. Mean ± s.e.m. values for each of these measurements, except the number of responders (n), was determined by averaging the values from all ROIs that reached the responder criterion. Note that in some experiments examining tachyphylaxis, a first stimulus with CAPS was used to identify neurons meeting the responder criterium, and measurements of Max. (F − F_0_)/F_0_ were repeated in the same cells for responses to a second stimulus including values even if these were below threshold for the latter. Every recording was performed on a single ganglion and in all cases replicates (N) were obtained from separate animals and experiments performed on different days. Student’s t-tests were performed in Microsoft Excel for Office 365. All data were plotted using GraphPad Prism 9.

## 5. Conclusions

A hyper-sensitive sub-population of CAPS-excitable neurons has been identified due to their independent and direct excitation by NGF. These neurons were sensitised even further by acute exposure to the neurotrophin. Our findings suggest that relatively low concentrations of NGF modulate the noxious sensitivity of a receptive subset of nociceptors in healthy tissues, in addition to the more established role of elevated NGF levels in inflammatory hyperalgesia.

## Figures and Tables

**Table 1:** Quantification of DRGNs excited by sequential exposure to CAPS and NGF.

		Number of Neurons Excited by the Reference Stimulus and Exposure to ^3^:-					
Cells Activated by Reference Stimulus ^1,2^		1 μM CAPS [B]	100 ng/mL NGF	0.3 μM CAPS [A]	1 μM CAPS [A]	1 μM CAPS [A] or [B] ^5^	Only Excited by the Reference Stimulus
0.3 µM CAPS [B] ^4^	28	23 (82)	13 (46)	9 (32)	20 (71)	26 (93)	1 (4)
1 µM CAPS [B] ^4^	234	-	75 (32)	20 (9)	195 (83)	234 (100)	28 (12)
100 ng/mL NGF (20 min)	84	-	-	18 (21)	76 (90)	81 (96)	2 (2) ^6^
0.3 µM CAPS [A] ^4^	28	-	-	-	24 (86)	26 (93)	2 (7)
1 µM CAPS [A] ^4^	219	-	-	-	-	219 (100)	14 (6)

^1^ Stimuli were applied to DRG as indicated in Figure 1A. CAPS was applied for 5 min. and NGF for 20 min. ^2^ A count of the neurons that displayed (within 20 min of stimulus application) an increase in fluorescence greater than 10 s.d. above a baseline level measured over a 2 min. period immediately prior to the application of stimulus. ^3^ Figures in parenthesis indicate cell numbers expressed as a % of the total activated by the reference stimulus. ^4^ [B] and [A] indicate, respectively, whether stimuli were applied before or after the DRG were exposed to NGF. ^5^ A count of all neurons excited by the reference stimulus and at least once by 1 μM CAPS out of two applications. A total of 262 unique cells were activated at least once during the two stimulation periods. ^6^ 2 cells were excited by NGF but neither 0.3 nor 1 μM CAPS; 3 cells (4% of 84) were excited by NGF but not 1 μM CAPS.

**Table 2:** Exposure to NGF for 20 min excites CAPS-sensitive neurons if applied to DRG before the vanilloid, and repeated exposure activates even more.

		Number of Neurons Excited by the Reference Stimulus and Exposure to ^3^: -					
Cells Activated by Reference Stimulus ^1,2^		1 μM CAPS [C1]	100 ng/mL NGF [N2]	1 μM CAPS [C2]	1 μM CAPS [C1 or C2] ^4^	10 μM CAPS [C3]	Only Excited by the Reference Stimulus
100 ng/mL NGF [N1] ^5^	57	47 (82)	21 (37)	35 (61)	49 (86)	48 (84)	3 (5)
1 μM CAPS [C1] ^5^	135	-	50 (37)	80 (59)	135 (100)	116 (86)	6 (4)
100 ng/mL NGF [N2] ^5^	72	-	-	48 (67)	60 (83)	58 (81)	5 (7)
1 μM CAPS [C2] ^5^	121	-	-	-	121 (100)	121 (100)	20 (17)
10 μM CAPS [C3] ^5^	251	-	-	-	132 (53)	-	107 (43)
100 ng/mL NGF [N1 or N2] ^5,6^	108	76 (70)	72 (67)	69 (64)	88 (81)	87 (81)	5 (5)

^1^ Stimuli were applied to DRG as indicated in Figure 6A. CAPS was applied for 5 min and NGF for 20 min. ^2^ A count of the neurons that displayed (within 20 min of stimulus application) an increase in fluorescence greater than 10 s.d. above a baseline level, measured over a 2 min period immediately prior to the application of stimulus. ^3^ Figures in parenthesis indicate cell numbers expressed as a % of the total activated by the reference stimulus. ^4^ A count of cells activated by the reference stimulus and 1 μM CAPS at least once during period [C1] or [C2]. ^5^ Labels in square brackets as defined in Figure 6 legend. ^6^ A count of cells activated at least once by NGF during period [N1] or [N2].

## Data Availability

The data presented in this study are available in the Supplementary Materials.

## Supplementary Materials

The following are available online at https://www.mdpi.com/1422-0067/22/5/2589/s1. Figure S1: Example traces of changes in fluorescence plotted against time for selected individual DRGNs., Table S1: Statistical analysis of data plotted in Figure 1C, Table S2: Statistical analysis of data plotted as a ratio in Figure 2B, Table S3: Statistical analysis of data plotted as a ratio in Figure 2C, Table S4: Statistical analysis of data plotted as a ratio in Figure 2D, Table S5: Statistical analysis of data in Figure 3B–D; comparisons between NGF-excitable and -refractory neurons., Table S6: Statistical analysis of part of the data in Figure 3B–D; comparisons between signals evoked by 1 μM CAPS applied before and after NGF in neurons that were excited both times., Table S7: Statistical analysis of part of the data in Figure 4A–C; comparisons between signals evoked before and after NGF, Table S8: Statistical analysis of data in Figure 5A–C; comparisons between signals evoked by 1 μM CAPS applied before and after NGF in neurons that were excited both times., Table S9: Statistical analysis of part of data in Figure 6C–E; comparisons between signals evoked in NGF-refractory cells and those excited once by NGF., Table S10: Statistical analysis of part of data in Figure 6C–E; comparisons between CAPS evoked signals in cells excited once by NGF with those activated twice.

## Abbreviations

aCSF	Artificial cerebrospinal fluid
aCSF-BOC	Carboxygenated aCSF containing bovine serum albumin
[A]	After NGF
[B]	Before NGF
[Ca^2+^]_i_	Intracellular Ca^2+^ concentration
CAPS	Capsaicin
DRG	Dorsal root ganglia
DRGNs	DRG neurons
NGF	Nerve growth factor
ROI	Region of interest
s.d.	Standard deviation
s.e.m.	Standard error of the mean
SNAP-25	Synaptosomal-associated protein of 25 kDa
TG	Trigeminal ganglia
TNFα	Tumour necrosis factor alpha
TrkA	Tropomyosin kinase receptor A
TRPA1	TRP ankyrin family member 1
TRPV1	Transient receptor potential vanilloid 1
