# Supplementary material for: Ca2+ Signalling Induced by NGF Identifies a Subset of Capsaicin-Excitable Neurons Displaying Enhanced Chemo-Nociception in Dorsal Root Ganglion Explants from Adult pirt-GCaMP3 Mouse
Source: Int J Mol Sci. 2021 Mar 4;22(5):2589. doi: 10.3390/ijms22052589 (PMC7961361; doi:10.3390/ijms22052589)
Supplement: Supplementary file 1 [file ijms-22-02589-s001.pdf]

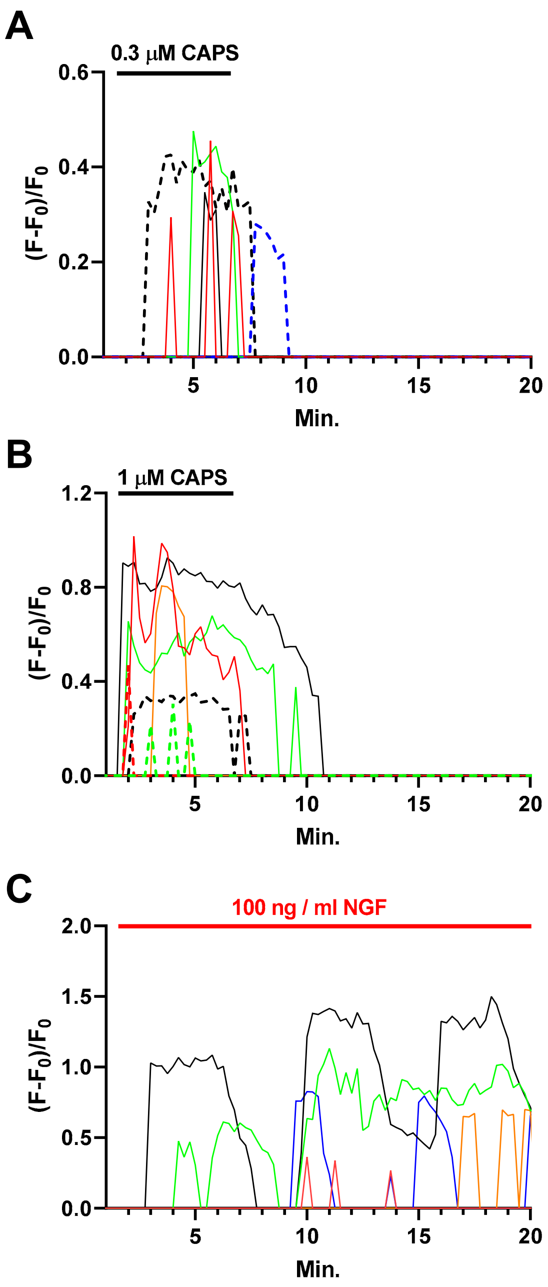

Supplementary Figure S1. Example traces of changes in fluorescence plotted against time for selected individual DRGNs. (**A**) and (**B**) show signals evoked by 0.3 and 1 mM CAPS, respectively, applied before NGF. Solid lines are taken from cells that were also excited during a subsequent 20 min. exposure to NGF (**C**), whilst broken lines in (**A**) and (**B**) indicate cells that did not respond to NGF. The solid red, green and black lines in (**A**), (**B**) and (**C**) represent repeat recordings from DRGNs that were excited by all three stimuli. Note that, for clarity, CAPS-evoked signals are not shown in (**A**) or (**B**) for NGF-responsive neurons indicated by the orange and blue traces in (**C**). The signals elicited by NGF typically fluctuated from high to low intensity for an extended period. Responses to CAPS were more variable; some cells displayed a single signal, others fluctuated, and many exhibited a single long signal followed by a period of fluctuation.

Supplementary Table S1. Statistical analysis of data plotted in Fig. 1C.

Group	5 min. NGF Mean ± s.e.m. (n)	20 min. NGF Mean ± s.e.m. (n)	Results of Student's two-tailed T- test Type [1]	dF [2]	t	P [4]
0.3 mM CAPS [B]	19±6 (3)	9±4 (3)	U	4	-1.34	0.25
1 mM CAPS [B]	79±8 (3)	78±23 (3)	U	2	-0.03	0.98
100 ng/ml NGF	6±4 (3)	28±5 (3)	U	4	3.62	**0.01**
0.3 mM CAPS [A]	24±6 (3)	9±3 (3)	U	3	-2.17	0.12
1 mM CAPS [A]	84±9 (3)	74±22 (3)	U	3	-2.35	0.71

[1] U, unpaired t-test assuming unequal variance, two-tailed. [2] dF, degrees of freedom. [3] t, t-statistic. [4] P, probability value; values below 0.05 were considered significant and are highlighted in bold.

Supplementary Table S2. Statistical analysis of data plotted as a ratio in Fig. 2B.

Group[2]	[CAPS] (mM)	Sets	Before NGF Mean ±s.e.m. (n)	After NGF Mean ± s.e.m. (n)	Results of Student's two-tailed T- test[1]			
					Type[5]	dF	T	P
Control	0.3	[B][3]	0.47±0.05 (14)	0.19±0.05 (14)	P	13	-4.72	**0.0004**
5 min NGF	0.3	[B][3]	0.52±0.03 (58)	0.40±0.05 (58)	P	57	-2.62	**0.01**
20 min NGF	0.3	[B][3]	0.41±0.04 (28)	0.22±0.05 (28)	P	27	-3.02	**0.006**
Control	1	[B][4]	0.56±0.03 (54)	0.24±0.03 (54)	P	53	-10.02	**<0.0001**
5 min NGF	1	[B][4]	0.63±0.02 (236)	0.66±0.03 (236)	P	235	1.06	0.28
20 min NGF	1	[B][4]	0.63±0.02 (234)	0.61±0.02(234)	P	233	-1.81	0.07

[1] In all cases, Max. (F-F_0)/F_0 induced by same concentration of CAPS were compared before and after NGF. Probability values below 0.05 were considered significant and are highlighted in bold. [2] Control group (N=1) was washed for 20 min. with aCSF lacking NGF. The 5 min. NGF group (N=3) was washed afterwards for 15 min. with aCSF. The 20 min. NGF group (N=3) was washed afterwards for 5 min. with aCSF. [3] Neurons that signalled above threshold when 0.3 mM CAPS was applied before NGF. Comparisons with values in the same cells the second time 0.3 mM CAPS was used after NGF, even if the latter were below threshold. [4] Neurons that signalled above threshold when 1 mM CAPS was applied before NGF. Comparisons were made with values observed in the same cells the second time 1 mM CAPS was used, even if the latter were below threshold. [5] P, paired, two-tailed.

Supplementary Table S3. Statistical analysis of data plotted as a ratio in Fig. 2C.

Group	[CAPS] (mM)	Sets	Before NGF Mean ± s.e.m. (n)	After NGF Mean ± s.e.m. (n)	Results of Student's two-tailed T- test[1]			
					Type[4]	dF	T	P
Control	0.3	[B]∩[A][2]	0.54±0.09 (3)	0.51±0.08 (3)	P	2	-3.73	0.065
5 min NGF	0.3	[B]∩[A][2]	0.57±0.04 (29)	0.67±0.06 (29)	P	28	1.47	0.15
20 min NGF	0.3	[B]∩[A][2]	0.39±0.07 (9)	0.49±0.09 (9)	P	8	0.85	0.42
Control	1	[B]∩[A][3]	0.65±0.05 (16)	0.55±0.05 (16)	P	15	-2.61	**0.02**
5 min NGF	1	[B]∩[A][3]	0.67±0.03 (183)	0.8±0.03 (183)	P	182	5.48	**<0.0001**
20 min NGF	1	[B]∩[A][3]	0.67±0.02 (195)	0.69±0.02 (195)	P	194	1.03	0.30

[1] In all cases, Max. (F-F_0)/F_0 induced by same concentration of CAPS were compared before and after NGF. Probability values below 0.05 were considered significant and are highlighted in bold. [2] Comparisons made only in neurons that signalled above threshold both times 0.3 mM CAPS was applied. [3] Comparisons made only in neurons that signalled above threshold both times 1 mM CAPS was applied. [4] P, paired, two-tailed.

Supplementary Table S4. Statistical analysis of data plotted as a ratio in Fig. 2D.

Group	[CAPS] (mM)	Sets	Before NGF Mean ± s.e.m. (n)	After NGF Mean ± s.e.m. (n)	Results of Student's two-tailed T- test			
					Type[3]	dF	T	P
Control	0.3	[B]v[A][1]	0.41±0.05 (17)	0.29±0.05 (17)	P	16	-2.19	**0.04**
5 min NGF	0.3	[B]v[A][1]	0.36±0.05 (102)	0.46±0.07 (102)	P	101	2.35	**0.02**
20 min NGF	0.3	[B]v[A][1]	0.33±0.03 (47)	0.33±0.04 (47)	P	46	-0.05	0.96
Control	1	[B]v[A][2]	0.53±0.03 (57)	0.27±0.03 (57)	P	56	-7.53	**<0.0001**
5 min NGF	1	[B]v[A][2]	0.53±0.04 (306)	0.64±0.04 (306)	P	305	4.42	**<0.0001**
20 min NGF	1	[B]v[A][2]	0.63±0.02 (259)	0.65±0.02 (259)	P	258	-1.05	0.30

[1] The Max. (F-F_0)/F_0 of all signals evoked before NGF compared with all signals elicited after in DRGNs that responded above threshold at least once out of two stimulations with 0.3 mM CAPS. Note that for cells that responded once only, below threshold values for the other stimulation were included in the analysis. [2] The Max. (F-F_0)/F_0 of all signals evoked before NGF compared with all signals after in DRGNs that responded at least once out of two stimulations with 1 mM CAPS. Note that for cells that responded once only, below threshold values for the other stimulation were included in the analysis. [3] P, paired, two-tailed.

Supplementary Table S5. Statistical analysis of data in Fig. 3B, C and D; comparisons between NGF-excitable and -refractory neurons.

Measure	[CAPS] (mM)	Sets [1]	NGF-refractory Mean ± s.e.m. (n)	NGF-excitable Mean ± s.e.m. (n)	Results of Student's two-tailed T- test Type [2]	dF	T	P [3]
Lag	0.3	[B]	9.8±1.6 (15)	6.5±1.7 (13)	U	26	-1.45	0.16
Lag	0.3	[A]	2.8±0.9 (10)	2.5±0.7 (18)	U	20	-0.25	0.8
Lag	1	[B]	1.9±0.2 (159)	1.0±0.1 (75)	U	232	-4.33	**<0.0001**
Lag	1	[A]	2.0±0.1 (144)	1.1±0.1 (76)	U	211	-4.74	**<0.0001**
Duration	0.3	[B]	4.1±1.1 (15)	3.7±1.0 (13)	U	26	-1.13	0.27
Duration	0.3	[A]	5.2±1.6 (10)	2.4±0.8 (18)	U	14	-1.12	0.28
Duration	1	[B]	4.0±0.3 (159)	5.8±0.3 (75)	U	177	4.06	**<0.0001**
Duration	1	[A]	3.9±0.2 (144)	6.6±0.4 (76)	U	123	5.49	**<0.0001**
Max. $(F-F_0)/F_0$	0.3	[B]	0.40±0.05 (15)	0.43±0.06 (13)	U	24	0.43	0.67
Max. $(F-F_0)/F_0$	0.3	[A]	0.43±0.06 (10)	0.51±0.06 (18)	U	24	0.84	0.41
Max. $(F-F_0)/F_0$	1	[B]	0.57±0.02 (159)	0.75±0.04 (75)	U	121	4.30	**<0.0001**
Max. $(F-F_0)/F_0$	1	[A]	0.66±0.03 (144)	0.77±0.04 (75)	U	125	3.45	**0.0008**

[1] Measurements made for above threshold signals evoked by the indicated concentrations of CAPS applied before [B] or after [A] NGF. [2] U, unpaired, unequal variance, two-tailed. [3] Probability values below 0.05 were considered significant and are highlighted in bold.

Supplementary Table S6. Statistical analysis of part of the data in Fig. 3B, C and D; comparisons between signals evoked by 1 mM CAPS applied before and after NGF in neurons that were excited both times.

Measure	[CAPS] (mM)	Sets [1]	Before NGF Mean ± s.e.m. (n)	After NGF Mean ± s.e.m. (n)	Results of Student's two-tailed T- test Type [2]	dF	T	P [3]
Lag	1	E	0.9±0.1 (69)	1.0±0.1 (69)	P	68	0.81	0.42
Lag	1	R	1.7±0.2 (125)	1.7±0.1 (125)	P	124	-0.19	0.85
Duration	1	E	6.0±0.3 (69)	6.8±0.5 (69)	P	68	2.86	**0.006**
Duration	1	R	4.0±0.3 (159)	3.9±0.2 (144)	P	125	-0.98	0.32
Max. $(F-F_0)/F_0$	1	E	0.77±0.04 (69)	0.80±0.04 (69)	P	68	1.18	0.24
Max. $(F-F_0)/F_0$	1	R	0.62±0.02 (125)	0.63±0.03 (125)	P	125	0.86	0.39

[1]E, NGF-excitable; R, NGF-refractory. [2] P, Paired, two-tailed. [3] Probability values below 0.05 were considered significant and are highlighted in bold.

Supplementary Table S7. Statistical analysis of part of the data in Fig. 4A, B, C; comparisons between signals evoked before and after NGF.

Measure	[CAPS] (mM)	Before NGF Mean ± s.e.m. (n)	After NGF Mean ± s.e.m. (n)	Results of Student's two-tailed T- test Type [1]	dF	T	P [2]
Max. $(F-F_0)/F_0$	0.3	0.52±0.03 (57)	0.59±0.04 (72)	U	124	1.38	0.17
Max. $(F-F_0)/F_0$	1	0.63±0.02 (236)	0.73±0.02 (253)	U	482	3.05	**0.002**
Duration	0.3	2.4±0.3 (57)	2.4±0.3 (72)	U	126	-0.01	0.99
Duration	1	3.0±0.2 (236)	3.6±0.2 (253)	U	476	2.18	**0.03**
Lag	0.3	6.0±0.7 (57)	4.0±0.5 (72)	U	114	-2.41	**0.01**
Lag	1	1.8±0.1 (236)	2.0±0.1 (253)	U	421	1.97	0.24

[1] P, Paired, two-tailed. [2] Probability values below 0.05 were considered significant and are highlighted in bold.

Supplementary Table S8. Statistical analysis of data in Fig. 5A, B and C; comparisons between signals evoked by 1 mM CAPS applied before and after NGF in neurons that were excited both times.

Measure	[CAPS] (mM)	Sets [1]	Before NGF Mean ± s.e.m. (n)	After NGF Mean ± s.e.m. (n)	Results of Student's two-tailed T- test Type [2]	dF	T	P [3]
Max. $(F-F_0)/F_0$	1	E	0.77±0.10 (13)	1.12±0.14 (13)	P	12	2.68	**0.02**
Max. $(F-F_0)/F_0$	1	R	0.65±0.03 (170)	0.78±0.03 (170)	P	169	4.89	**<0.0001**
Duration	1	E	4.7±0.7 (13)	6.2±0.8 (13)	P	12	2.42	**0.03**
Duration	1	R	3.2±0.2 (170)	4.0±0.2 (170)	P	169	4.42	**<0.0001**
Lag	1	E	1.3±0.4 (13)	1.2±0.3 (13)	P	12	0.82	0.62
Lag	1	R	1.7±0.1 (170)	1.6±0.1 (170)	P	169	-0.76	0.45

[1] E, NGF excitable; R, NGF refractory. [2] P, Paired, two-tailed. [3] Probability values below 0.05 were considered significant and are highlighted in bold.

Supplementary Table S9. Statistical analysis of part of data in Fig. 6C, D and E; comparisons between signals evoked in NGF-refractory cells and those excited once by NGF.

Measure	[CAPS] (mM)	NGF-refractory Mean ± s.e.m. (n)	Excited once by NGF Mean ± s.e.m. (n)	Results of Student's two-tailed T- test Type [1]	dF	T	P [2]
Lag	1 (C1) [3]	4.2±0.6 (59)	2.2±0.2 (55)	U	76	-3.17	**0.002**
Lag	1 (C2) [3]	4.1±0.5 (51)	2.1±0.2 (55)	U	69	-3.77	**0.0003**
Lag	10 (C3) [3]	2.5±0.2 (164)	1.2±0.2 (67)	U	198	-4.15	**<0.0001**
Duration	1 (C1) [3]	2.3±0.3 (59)	4.4±0.5 (55)	U	90	3.47	**0.0008**
Duration	1 (C2) [3]	2.6±0.3 (51)	4.5±0.4 (55)	U	104	3.66	**0.0004**
Duration	10 (C3) [3]	7.2±0.4 (164)	9.8±0.8 (68)	U	104	2.83	**0.006**
Max. $(F-F_0)/F_0$	1 (C1) [3]	0.45±0.04 (59)	0.51±0.02 (55)	U	101	1.26	0.21
Max. $(F-F_0)/F_0$	1 (C2) [3]	0.46±0.06 (51)	0.44±0.02 (51)	U	65	-0.28	0.78
Max. $(F-F_0)/F_0$	10 (C3) [3]	0.62±0.02 (164)	0.61±0.04 (68)	U	115	-0.15	0.88

[1] U, Unpaired, unequal variance, two-tailed. [2] Probability values below 0.05 were considered significant and are highlighted in bold. [3] Labels within brackets are defined in Fig. 6 Legend.

Supplementary Table S10. Statistical analysis of part of data in Fig. 6C, D and E; comparisons between CAPS evoked signals in cells excited once by NGF with those activated twice.

Measure	[CAPS] (mM)	Excited once by NGF Mean ± s.e.m. (n)	Excited twice by NGF Mean ± s.e.m. (n)	Results of Student's two-tailed T- test Type [1]	dF	T	P [2]
Lag	1 (C1) [3]	2.2±0.2 (55)	0.8±0.2 (21)	U	72	-5.09	**<0.0001**
Lag	1 (C2) [3]	2.1±0.2 (55)	1.0±0.2 (14)	U	50	-4.04	**0.0002**
Lag	10 (C3) [3]	1.2±0.2 (67)	0.27±0.1 (19)	U	76	-4.17	**<0.0001**
Duration	1 (C1) [3]	4.4±0.5 (55)	6.7±0.8 (21)	U	40	2.53	**0.02**
Duration	1 (C2) [3]	4.5±0.4 (55)	6.0±1.0 (14)	U	17	1.44	0.17
Duration	10 (C3) [3]	9.8±0.8 (68)	11.6± 1.3 (19)	U	33	1.19	0.24
Max. $(F-F_0)/F_0$	1 (C1) [3]	0.51±0.02 (55)	0.56±0.05 (21)	U	30	1.02	0.31
Max. $(F-F_0)/F_0$	1 (C2) [3]	0.44±0.02 (51)	0.53±0.05 (14)	U	19	1.61	0.12
Max. $(F-F_0)/F_0$	10 (C3) [3]	0.61±0.04 (68)	0.56±0.05 (19)	U	39	-0.79	0.21

[1] U, Unpaired, unequal variance, two-tailed. [2] Probability values below 0.05 were considered significant and are highlighted in bold. [3] Labels within brackets are defined in Fig. 6 Legend.
